# Gelatin Hydrogel pH Sensors Incorporating Anthocyanins for Intelligent Food Packaging: Towards Enhanced Food Spoilage Detection

**DOI:** 10.3390/gels12040292

**Published:** 2026-03-31

**Authors:** Pompilia Mioara Lopes, Liviu Mare, Lucian Barbu-Tudoran, Alina Gadja, Violeta Popescu

**Affiliations:** 1 Physics and Chemistry Department, Technical University of Cluj-Napoca, 28 Memorandumului Str., 400114 Cluj-Napoca, Romania; mioara.lopes@im.utcluj.ro (P.M.L.); mareliviumarius@gmail.com (L.M.); alina.gadja@campus.utcluj.ro (A.G.); 2 Electron Microscopy Center, Faculty of Biology and Geology, Babeș-Bolyai University of Cluj-Napoca, 1 M. Kogălniceanu Street, 400347 Cluj-Napoca, Romania; lucian.barbu@ubbcluj.ro

**Keywords:** whey-and-gelatin-based hydrogel, red cabbage extract, pH sensor, food packaging

## Abstract

Protein-based hydrogels composed of gelatin, whey and glycerol were functionalized with red cabbage extract (RCE) to develop natural colorimetric pH sensors for intelligent food packaging. Structural analysis by X-ray diffraction (XRD) and scanning electron microscopy (SEM) revealed amorphous, hierarchically organized networks where RCE molecules interact with protein chains. The resulting microstructure, consisting of compact surface domains and a porous internal network, may regulate the diffusion of volatile amines into the hydrogel matrix, enabling gradual and stable pH-dependent color transitions. The resulting biocomposite hydrogel exhibited a stable and time-resolved optical response to meat spoilage, correlating structural relaxation with colorimetric sensitivity. Color difference values (ΔE_00_) calculated based on recorded images indicated strong chromatic changes in the presence of spoilage-related volatiles. Under refrigeration, ΔE_00_ remained below five, suggesting negligible color shifts. At room temperature, ΔE_00_ exceeded 20 after 48 h, confirming significant anthocyanin transformation linked to increased alkalinity (pH 7.2–7.5). A positive correlation between ΔE_00_ and pH was observed, highlighting the hydrogel’s high sensitivity to environmental changes. These findings confirm the potential of RCE-loaded hydrogels as eco-friendly, visual freshness indicators suitable for intelligent packaging applications. The hydrogel films demonstrated a distinct color transition within the pH range of 5.75–7.5, corresponding to the freshness variation interval of chicken meat.

## 1. Introduction

The spoilage of meat products, particularly poultry, is closely associated with microbial activity that induces the release of volatile compounds such as ammonia (NH_3_) [1,2], carbon dioxide (CO_2_) [3], and hydrogen sulfide (H_2_S) [2,3] through biochemical processes including decarboxylation [1], deamination, and desulfurization of amino acids [1,2,3]. These metabolites cause pH shifts [1] that can serve as direct indicators of meat spoilage. In this regard, various pH-sensitive dyes have been used in intelligent packaging systems to provide a visual indication of spoilage [4,5,6,7].

Despite the advancements in synthetic materials research, a critical gap persists in the literature concerning the toxicological safety and long-term effects of such materials in direct or indirect contact with food. This underlines the need to develop safer, non-toxic, and biodegradable alternatives for real-time freshness monitoring in meat packaging [8].

The integration of biopolymers and naturally derived colorimetric sensors into food packaging systems [9,10] represent a promising direction in the development of intelligent packaging, due to their economic and environmental advantages. The use of renewable resources, the biodegradability of the materials, and their potential to reduce the carbon footprint support the sustainability of such packaging solutions.

In recent years, there has been an increasing interest in developing eco-friendly and biocompatible chromogenic sensors for monitoring food quality and safety [8,9]. Among natural pigments, anthocyanins represent a versatile class of natural colorants capable of exhibiting distinct color changes across a wide pH range, corresponding closely to the biochemical transformations associated with meat spoilage. Numerous studies have focused on valorizing anthocyanins extracted from various food sources and by-products of the food industry, such as red cabbage [11,12], grape skins [13], purple sweet potato [9,14], and roselle calyx [15], as intelligent freshness indicators for food packaging applications. These natural pigments are highly sensitive to ambient pH variations, easy to interpret visually, cost-effective, and compatible with sustainable production methods [6,16,17]. As phenolic compounds belonging to the flavonoid family, anthocyanins are widespread in plants—from roots to aerial parts—imparting vivid red, purple, and blue hues and are readily extractable in aqueous solvents using simple, eco-compatible techniques [18,19].

The incorporation of anthocyanins into natural polymeric matrices, particularly carbohydrate-based systems such as cellulose nanocrystals and tara gum [13], bacterial cellulose [11], and chitosan [20], or protein-based systems [7,21,22,23] such as whey protein and gelatin [6,22,23,24,25,26], has been widely investigated to develop smart biopolymer composites. Recent advancements in gelatin-based hydrogel systems incorporating anthocyanins, notably from *Hibiscus sabdariffa*, have demonstrated rapid vapor-induced chromatic transitions, with distinct color changes observed within minutes of ammonia [7]. These findings emphasize the inherent structural compatibility between protein matrices and anthocyanins, which underpins the efficiency and reproducibility of colorimetric sensing platforms.

Previous studies [27,28,29,30] conducted by the authors have demonstrated that the combination of whey protein and gelatin leads to the formation of hydrogels with enhanced elasticity and superior water retention capacity, due to the role of whey proteins as co-gelling agents that stabilize the three-dimensional gelatin network. In these earlier works, the whey–gelatin polymeric matrix was investigated in various formulations, including combinations with cross-linking agents such as copper sulfate [27,31] and graphene oxide [28,29], with the common objective of developing biodegradable and non-toxic materials suitable for food packaging applications.

Building upon these insights, our previous results on a gelatin-whey protein hydrogel formulation demonstrated enhanced elasticity, superior water retention capacity, and preserved structural integrity—properties that rendered this system particularly suitable for use in the present study. Therefore, these characteristics were further exploited in the formulation and evaluation of a pH-responsive gelatin–whey hydrogel enriched with red cabbage extract (RCE), designed to function as a colorimetric indicator for non-contact monitoring of meat spoilage [32]. For sample characterization, three hydrogel variants were investigated: a control hydrogel containing whey, gelatin, and glycerol without RCE (WGeGly), a hydrogel incorporating RCE dried at 25 °C (WGRCE), and a thermally treated hydrogel (WGRCEt). Structural (XRD) and colorimetric analyses were carried out to correlate the internal organization and surface morphology (SEM) of the hydrogels with their optical and pH-responsive behavior, thereby elucidating the mechanisms underlying their sensing performance.

## 2. Results and Discussion

The results are organized to provide a comprehensive view of the structural, morphological, and functional characteristics of the developed gelatin–whey hydrogel systems. The first part of the study focuses on evaluating the colorimetric pH sensitivity of the hydrogel containing RCE under a real storage condition, followed by detailed microstructural (SEM), and consequently a crystallographic (XRD) characterization of the three hydrogel variants (WGeGly, WGRCE, WGRCEt) and their precursors. This organization enables the correlation of macroscopic color changes with the internal structural features responsible for the sensor’s optical performance.

### 2.1. Assessment of pH Responsive Gelatin–Whey Hydrogel Containing Red Cabbage Extract (RCE) for Chicken Meat Spoilage Detection

To evaluate the practical sensing performance of the hydrogel indicator, its colorimetric response was investigated under real food storage conditions. The experiment was designed to assess the hydrogel’s colorimetric reaction to volatile compounds released during chicken meat spoilage. Hydrogel patches were attached to the inner side of the chicken meat packages, allowing non-contact sensing of volatile amines and other basic gases. Six identical patches were prepared from the main WGRCE formulation (whey–gelatin–glycerol–red cabbage extract dried at 23 °C). These samples, labeled P_1_–P_6_, differed only in their storage conditions during the freshness test. Specifically, three hydrogel patches (P_1_–P_3_) were placed in chicken meat packages stored under refrigeration (4 °C), while the remaining three (P_4_–P_6_) were stored at room temperature (23 °C). This design enabled direct comparison of the hydrogel’s optical response to distinct spoilage rates under cold and ambient conditions.

The hydrogel patch demonstrated a clear pH-dependent color change associated with the spoilage progression of chicken meat under different storage conditions. For the samples stored at refrigeration temperature (4 °C), no visible color change was observed throughout the 132 h monitoring period. The hydrogel indicators retained their initial coloration, indicating minimal microbial growth and negligible volatile amine formation during cold storage. This behavior is consistent with previous studies reporting on anthocyanin-based freshness indicators applied to refrigerated meat and fish systems. For instance, Postolovic et al. [33] observed that a carrageenan–anthocyanin/chitosan bilayer film exhibited no significant color change during the first six days of trout storage at 4 °C, confirming the film’s stability and lack of response under minimal spoilage conditions. Similarly, Badhury et al. [16] reported that a red cabbage-based nanocellulose indicator applied to beef packaging showed only minor color variation until day 5 of storage at 4 °C, after which color transitions became more pronounced due to amine accumulation. Comparable trends were also found by Zam et al. [34], who developed a gelatin–chitosan–CMC multilayer film incorporating RCE that remained pink during the early storage period and gradually shifted to blue, green, and finally yellow as nitrogen content increased in the package headspace between days 6 and 10 of fish storage.

**Figure 1:**
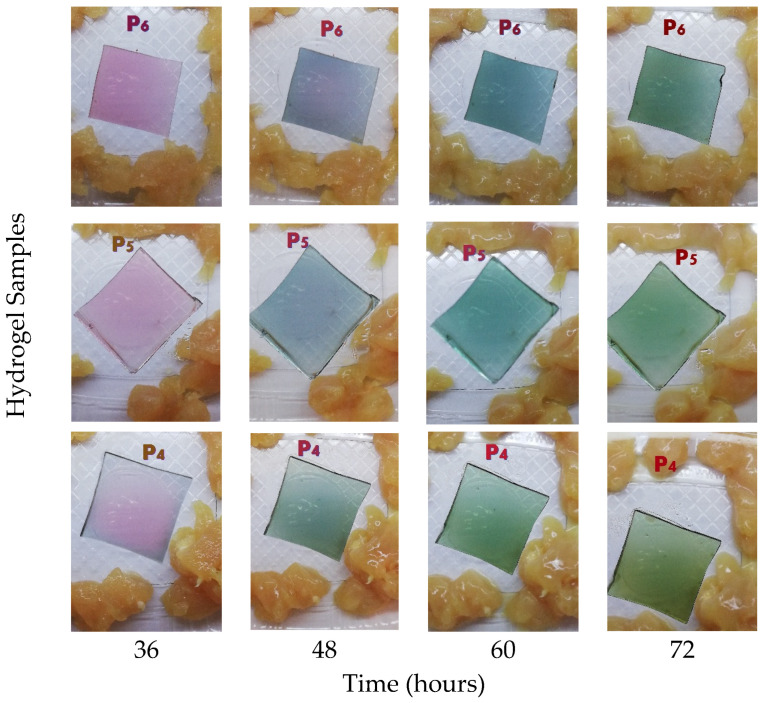
Colorimetric response of hydrogel indicators exposed to chicken meat during storage at room temperature: the variations in color intensity and distribution are attributed to the non-uniform diffusion of volatile amines within the package, with initial changes occurring at the edges of the hydrogel due to referential exposure to gaseous compounds released during meat spoilage.

In contrast, as can be seen in Figure 1, the samples stored at room temperature (23 °C) showed a gradual and distinct color change in the hydrogel films starting after 24 h of storage. This alteration was associated with protein degradation and the formation of basic volatile compounds such as ammonia and amines, which elevate the local pH [3]. After 48 h, visual observations suggested the onset of spoilage, and photographic documentation was discontinued due to extensive decomposition.

Our results confirm the hydrogel’s sensitivity to volatile amines typically associated with meat spoilage. The hydrogel films showed high stability under refrigeration and strong reactivity at ambient temperature, indicating their potential as non-invasive, real-time freshness indicators in intelligent food packaging systems.

### 2.2. Colorimetric Response of Red Cabbage Extract-Based Hydrogels to Meat Spoilage Gases

The degradation of meat products takes place at a higher rate at room temperature. Initial minor color variations observed in the refrigerated samples may be attributed to moisture absorption from the package headspace.

The results revealed marked differences between the samples stored at 4 °C and those maintained at room temperature (Figure S1 from Supplementary Material). At 4 °C, under refrigerated storage, the color changes were minimal (ΔE_00_ < 5). In contrast (at room temperature), the samples exhibited pronounced color shifts after 36–48 h, with ΔE_00_ values exceeding 20, indicating a strong interaction with volatile compounds released during meat spoilage.

The use of the ΔE_00_ metric ensured a perceptually relevant quantification of the observed color changes, allowing direct comparison between storage conditions and exposure times. The ΔE_00_ values obtained in this study fall within the range typically reported for anthocyanin-based spoilage indicators [15,18,33,35,36].

The use of the CIEDE2000 metric [36] ensured perceptually uniform quantification of color differences, which is particularly relevant for anthocyanin-based materials exhibiting nonlinear shifts across red–purple–blue domains.

**Figure 2:**
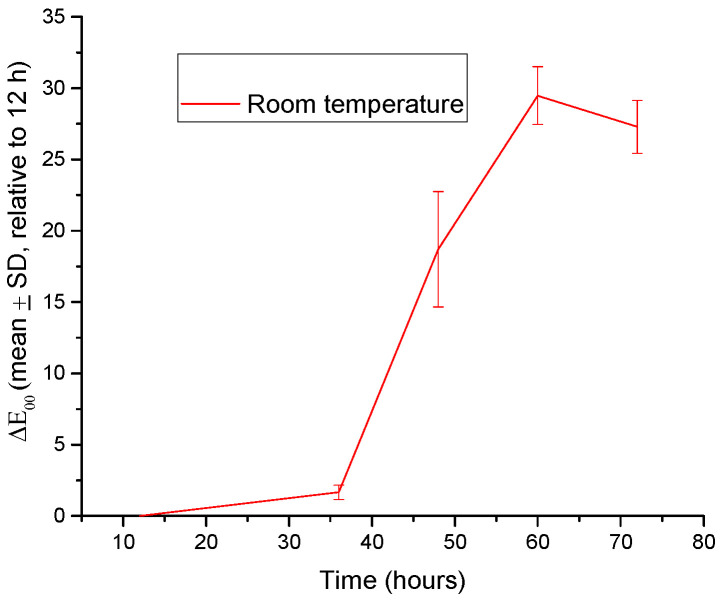
Evolution of the color difference (ΔE_00_, mean ± SD, *n* = 3) of red cabbage extract-loaded hydrogel samples exposed to chicken meat at room temperature (23 °C). Calculations were performed using the CIEDE2000 formula relative to the 12 h reference point, which represents the stabilized state of the hydrogel following initial moisture equilibration.

Due to the negligible color changes observed at 4 °C (ΔE_00_ < 5), only the results obtained at room temperature are presented in Figure 2.

**Figure 3:**
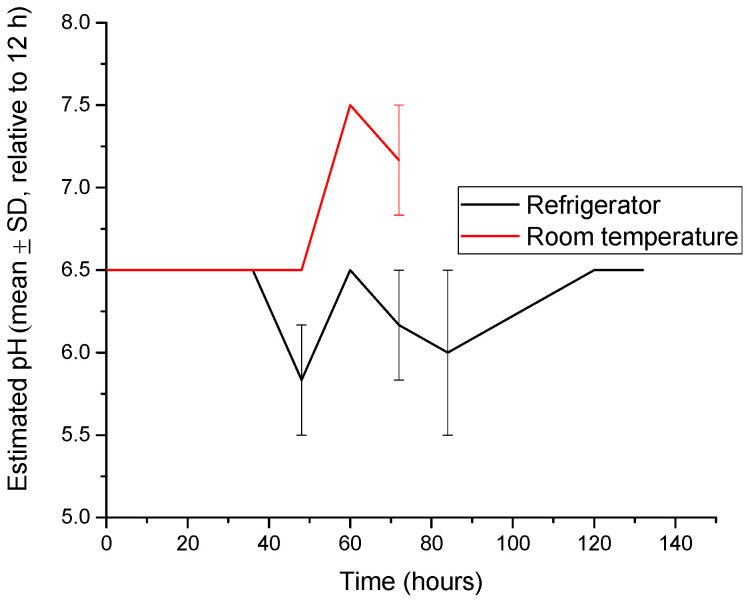
The evolution of the average value of the pH (mean ± SD, *n* = 3) for hydrogel samples in the presence of meat at room temperature (23 °C) and refrigerated (4 ± 1 °C).

Based on the calibrated colorimetric scale, the apparent pH of the hydrogel indicator environment remained stable between 5.2 and 6.0 under refrigerated conditions, whereas at room temperature conditions, the estimated pH increased progressively to 7.2–7.5. This behavior is associated with the accumulation of basic volatile compounds, such as ammonia and biogenic amines, generated during protein degradation in meat products (Figure 3) [16,37]. The progressive increase in the estimated pH observed under room temperature conditions is consistent with previous studies reporting the accumulation of basic volatile compounds, such as ammonia and biogenic amines, during protein degradation in meat and fish products [37]. Similar pH shifts toward neutral and alkaline values have been widely reported for anthocyanin-based intelligent packaging systems designed for freshness monitoring [18].

The observed pH increase led to pronounced chromatic changes in the anthocyanin-loaded hydrogels, consistent with the well-documented pH-dependent structural transformations of anthocyanins from flavylium cation forms to quinoidal base and chalcone structures under alkaline conditions [38,39,40]. A positive correlation was observed between the color difference (ΔE_00_) and the estimated pH, with increasing ΔE_00_ values accompanying the progressive alkalization of the environment. Similar correlations between chromatic response and pH variation have been reported for anthocyanin-based intelligent packaging systems designed for food freshness monitoring [12]. Visually perceptible color changes (ΔE_00_ > 5) corresponded to an approximate variation of 0.5–1 pH unit, indicating the high sensitivity of the hydrogel to pH changes [41]. This behavior is consistent with pH-dependent structural transformations of anthocyanins, which induce pronounced shifts in chromatic properties under alkaline conditions.

The reported pH values correspond to apparent pH values estimated from the colorimetric calibration curve of the hydrogel indicators, rather than direct electrochemical measurements of the meat matrix.

In this context, the development of biodegradable intelligent packaging systems based on renewable biopolymers and natural chromogenic compounds has emerged as an important strategy for improving food safety while reducing environmental impact. Such systems enable real-time visual monitoring of food freshness without opening the package, offering a simple and consumer-friendly approach for spoilage detection. Colorimetric indicators are particularly attractive because they allow rapid visual interpretation by consumers without the need for specialized instrumentation.

Future work will focus on correlating the colorimetric response of the hydrogel indicators with conventional spoilage markers such as total volatile basic nitrogen (TVB-N) and microbial counts, in order to establish quantitative freshness thresholds for practical food packaging applications.

### 2.3. Morphological Analysis by SEM

**Figure 4:**
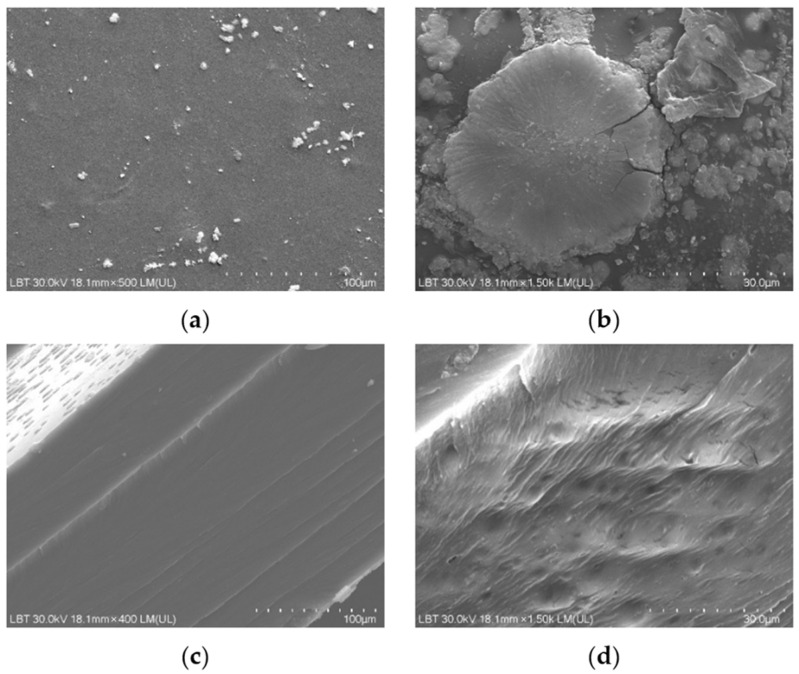
SEM micrographs illustrating the surface and cross-sectional morphology of gelatin–whey hydrogels incorporating red cabbage extract: (**a**) general view of the upper surface of the hydrogel sample (scale bar = 100 µm; magnification ≈ 500×), (**b**) surface localized whey protein aggregates with rosette-like and sponge-like morphology (30 µm; ≈1500×), (**c**) multilamellar architecture observed in the hydrogel cross-section (100 µm; ≈400×), (**d**) porous network structure evident in the hydrogel cross-section (30 µm; ≈1500×).

The first aspect examined by SEM analysis was the degree of uniformity of the samples and the dispersion and distribution of anthocyanins from the RCE within the protein matrix. As visually observed and shown in Figure 1, the hydrogel samples appeared smooth, without any visible agglomerates or surface irregularities to the naked eye. The high water solubility of RCE facilitated its uniform dispersion throughout the hydrogel network, without inducing noticeable changes in the overall surface topography [22,40]. This observation is consistent with previous reports on bacterial/cellulose nanofiber/gelatin-based intelligent films incorporating RCE, which also exhibited homogeneous surfaces, in contrast to similar films containing curcumin that displayed a rougher morphology due to limited pigment solubility and aggregation [35]. Further, surface SEM micrographs revealed the presence of compact rosette-like agglomerates (see Figure 4a,b), originating from the crystallization of whey proteins during the drying process, similar to the microstructural patterns reported by other authors [23] in carrageenan/gelatin–anthocyanin composite systems, where protein-polyphenol hydrogen bonding induced local ordering. Although such aggregates might initially appear as morphological imperfections that could hinder rapid gas interaction, they may in fact play a functional role. These protein-rich domains could act as a semi-permeable surface layer regulating the penetration rate of volatile amines. These protein-rich domains could function as a semi-permeable layer, modulating the penetration rate of volatile amines and delaying the chromatic response at the surface. This controlled delay enables the inner porous and lamellar regions of the hydrogel—clearly observed in cross-sectional SEM images (Figure 4c,d)—to absorb and retain spoilage gases more effectively [22]. Such an internal first response mechanism may provide a more stable and progressive color change, reducing false positives and better reflecting the actual spoilage state of the packaged meat. Therefore, this dual morphological configuration, combining a dense superficial barrier with a gas-permeable interior, may represent an advantageous feature for intelligent packaging systems requiring gradual, time-resolved freshness indicators.

Figure 4d shows the presence of an inner porous network structure in the hydrogel cross-section. Consequently, this layered morphology likely promotes gas penetration and retention, enabling a sustained and progressive colorimetric response—a highly desirable trait for freshness indicators designed for real-time monitoring in packaged meat systems. Previously reported SEM observations of gelatin-based films incorporating red cabbage anthocyanins [22] suggests structural compatibility between the protein network and the embedded pigment. The ability of gelatin to generate porous and hierarchically organized structures has also been extensively documented in aerogel systems. Yuan Wu et al. reported comparable diffusion-regulating behavior in gelatin–gum Arabic aerogels, where SEM analysis revealed dense outer layers combined with highly porous inner networks. This architecture enhanced gas retention and enabled gradual, stable colorimetric transitions during shrimp and pork spoilage [41].

The dual microstructure revealed by SEM, consisting of a compact surface layer and a porous internal network, may play an important role in regulating the diffusion of volatile amines into the hydrogel matrix. Such architecture could enable a more gradual and stable colorimetric response, which is advantageous for real-time freshness indicators used in intelligent food packaging.

### 2.4. Structural Analysis by XRD

**Figure 5:**
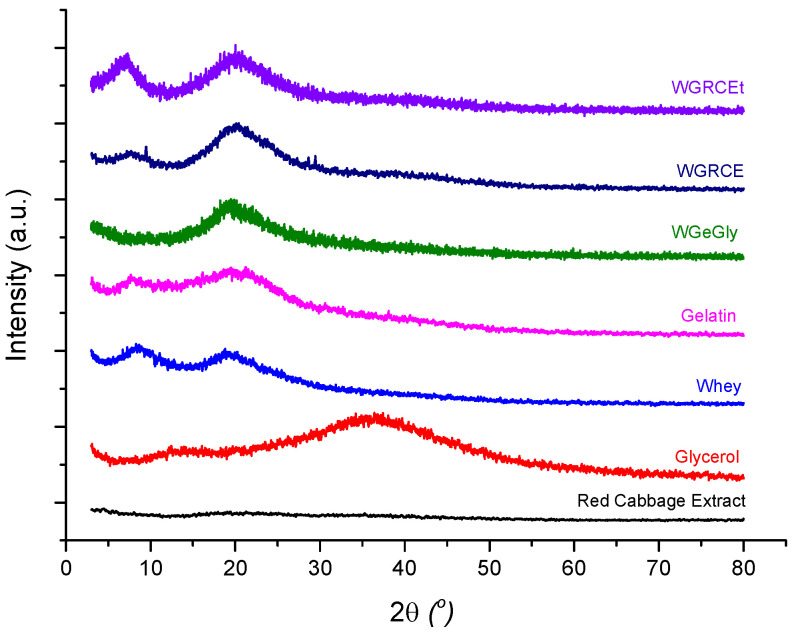
X-ray diffraction (XRD) patterns of the gelatin–whey-based hydrogels and their individual precursors (gelatin, whey, glycerol, and red cabbage extract—RCE), and the protein-based hydrogels: control hydrogel—WGeGly, hydrogel containing RCE dried at 25 °C—WGRCE, and thermally treated hydrogel—WGRCEt.

The XRD patterns of the precursors and of the protein-based hydrogels investigated in this study are presented in Figure 5, while the position of the main diffraction peaks, full width at half maximum (FWHM), and the degree of crystallinity are summarized in Table 1. In addition to the main WGRCE hydrogel formulation, two supplementary comparative samples were analyzed: the thermally treated hydrogel (WGRCEt), obtained by heating the initially dried hydrogel at 50 °C for 3 days, and the control hydrogel (WGeGly), containing only whey, gelatin, glycerol and distilled water. These two additional samples were included to assess the influence of red cabbage extract (RCE) and the subsequent heat treatment on the structural order of the protein network.

All XRD profiles exhibit two broad diffraction peaks, at approximately 2*θ* ≈ 7–8° and ≈19–20, typical of gelatin–whey systems with predominantly amorphous character and local semicrystalline ordering, respectively. FWHM values were determined for each sample, corresponding to the high-angle reflection (FWHM_1_), linked to β-sheet-type folding within short-range ordered domains, and the low-angle reflection (FWHM_2_) associated with helix-related scattering and intermolecular chain spacing.

Whey protein (W) exhibits two main peaks at 2*θ* ≈ 8.36° and 18.95°, consistent with semi-organized structures of β-lactoglobulin and α-lactalbumin. These reflections confirm locally ordered domains stabilized by hydrophobic interactions and hydrogen bonding. The reflection at 2*θ* ≈ 8.36° associated with the helical peak can be related to the characteristic periodicity of the helix with *d* = 10.56 Å, typical of helical structures of partially folded and denatured protein domains, while the one at 2*θ* ≈ 18.95° (*d* = 4.68 Å) suggests β-sheet arrangements, confirming the semicrystalline character of the dried whey. The degree of crystallinity is 40.50% higher than the crystallinity of gelatin, which is 10.90%, reflecting more helical domains in the whey protein matrix.

Gelatin (Ge) shows two broad diffraction peaks at 2*θ* ≈ 7.89° and 19.11°. The first reflection at the lower angle is a specific signature of the triple-helical structures (renatured collagen-like domains), and *d* reflects a characteristic distance related to the helical pitch of the polymer chains (*d* ≈ 11.44 Å) [28]. The second, more intense peak corresponds to the amorphous protein lattice and the β-sheet packing, with a mean spacing of about 4.64 Å between adjacent peptide chains linked by hydrogen bonds and Van der Waals forces [26].

The XRD pattern of pure glycerol is characterized by two main amorphous features. A primary broad reflection is observed at 2*θ* ≈ 36–37°, corresponding to a d-spacing of approximately 2.5 Å. According to Towey et al. (2011) [42], this region is associated with short-range atomic correlations, specifically oxygen–oxygen (O···O) within the extensive hydrogen-bonding network of the liquid. Additionally, a secondary halo at a lower angle distance (2*θ* ≈ 13° *d* ≈ 6.8 Å) represents the intermediate-range order, reflecting the average packing distance between glycerol molecular centers. The absence of sharp peaks confirms the completely amorphous, liquid state of the sample.

Red cabbage extract (RCE) exhibits a completely diffuse halo without distinct peaks, confirming its amorphous character [43]. The weak fluctuations in intensity correspond to transient density variations arising from the heterogeneous mixture of anthocyanins, flavonoids, phenolic acids, sugars, and bound water. Such an amorphous structure enables uniform dispersion of RCE within the protein matrix, improving pigment stabilization and overall homogeneity without introducing rigidity or crystalline phases.

In the absence of RCE, the control hydrogel (WGeGly) displays one reflection at 2*θ* ≈ 19.35°, corresponding to interplanar distances of 4.48 Å, respectively, indicating a poorly ordered lattice with limited alignment of protein chains, consistent with partial intermolecular organization between gelatin and whey.

The WGRCE hydrogel (dried at 23 °C) shows two main peaks at 2*θ* ≈ 7.5° and 20.22°, like those of the protein precursors but slightly shifted toward smaller angles. The shift suggests partial compaction of the polymeric lattice and specific interactions between protein chains and phenolic compounds from the extract. The first reflection (2*θ* ≈ 7.5°, *d ≈* 11.78 Å) is attributed to the triple-helical arrangement of the protein network, where the interchain spacing is increased by the intercalation of glycerol molecules within the gelatin–whey complexes. The second one (2*θ* ≈ 20.22°, *d* ≈ 4.39 Å) represents local β-sheet arrangements stabilized by short hydrogen bonds (O–H···O and C=O···H–N). The broad character of these peaks indicates local ordering within an amorphous lattice. Furthermore, the diffraction pattern of WGRCE suggests a subtle broadening in the ranges of 9–11° and 28–30°. While these features are near the detection limit, they might reflect localized, short-range molecular packing induced by the interaction between the protein matrix and the polyphenolic components during the equilibration process. Interestingly, reflections occurring in the same 2θ range (≈9° and ≈29°) were also identified in our previous study on graphene oxide (GO)-reinforced gelatin–whey hydrogels, and in mung bean protein hydrogel loaded with curcumin [44], suggesting that such structural motifs may represent a recurring organization pattern in protein-based lattices, regardless of the reinforcing or functional additive used [28].

The thermally treated hydrogel (WGRCEt, 50 °C, 3 days) was not included in the colorimetric or packaging tests but was analyzed by XRD for structural comparison purposes. The diffraction pattern exhibits peaks at 2*θ* ≈ 7.20° and 19.92°. The slight shift in the first peak from 2*θ* ≈ 7.5° (sample WGRCE) to 7.20° (sample WGRCEt) reflects an increase in interchain distances (*d* ≈ 12.27 Å) accompanied by a reduction in FWHM to 3.20°. This decrease in FWHM, compared to the untreated WGRCE (3.86°), clearly demonstrates that controlled thermal treatment facilitates a more efficient molecular rearrangement, leading to better-defined and more stable helical domains with an increase in the degree of crystallinity to 25.3% compared to 5.8%, the value obtained for WGRCE. The second reflection at 2*θ* ≈ 19.92° (*d* ≈ 4.45 Å) remains related to β-sheet packing and hydrogen-bonded regions. Furthermore, the crystallinity index calculation confirms this trend: the crystallinity decreased from 10.9% (Ge) to 5.8% upon RCE addition (WGRCE), but was successfully restored to 25.3% after thermal treatment (WGRCEt). These results suggest that while anthocyanins initially disrupt the gelatin’s self-assembly, the subsequent thermal energy allows the system to reach a more ordered and thermodynamically stable state. The second reflection around 2*θ* ≈ 20° remains consistent across samples, confirming that the overall amorphous framework remains stable while the local helical organization is significantly optimized by the treatment. Although the sample was not functionally tested, this analysis provides valuable insight into the structural evolution of the hydrogel lattice under thermal stress, supporting the interpretation that controlled heat exposure enhances homogeneity and increases the degree of crystallinity.

The narrower FWHM values of the small-angle diffraction peaks (2.92–3.86°), compared to those of the amorphous phase (6.8–11.4°), suggest a higher degree of structural order and partial crystallinity in the studied compounds.

### 2.5. FT-IR Structural Analysis

**Figure 6:**
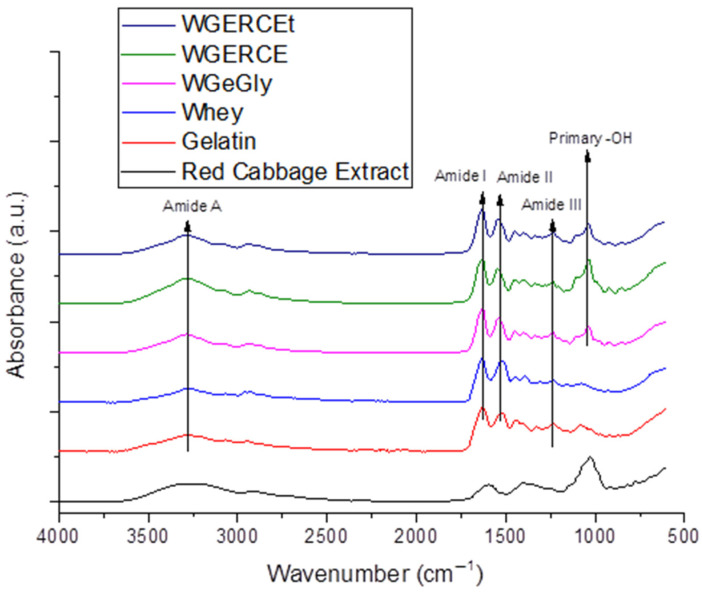
IR spectra of hydrogels based on whey and gelatin with RCE (WGRCE) and control samples (WGeGly) and their precursors, whey and gelatin.

The FT-IR spectra of red cabbage extract, gelatin, whey, WGeGly, and WGRCE samples are presented in Figure 6. All spectra exhibit a broad absorption band centered around 3287 cm^−1^, corresponding to the Amide A band, which is mainly associated with N–H stretching vibrations overlapped with O–H stretching from hydrogen-bonded hydroxyl groups [15,18,28]. The increased intensity of this band in the WGRCE sample suggests enhanced hydrogen bonding interactions induced by the presence of red cabbage extract, rich in polyphenolic compounds [26]. The decrease in the intensity of the amide A band after thermal treatment and the shift from 3295 cm^−1^ to 3293 cm^−1^ indicate a higher degree of structural organization compared to room-temperature drying. The concomitant decrease in the 1041 cm^−1^ peak intensity in the thermal-treated sample suggests that the primary –OH groups of glycerol are sequestered within the crystalline domains. This synergy between thermal energy and controlled evaporation promotes a more ‘ordered’ molecular assembly, where glycerol bridges the whey protein and gelatin chains, effectively enhancing the physical cross-linking density of the hydrogel matrix.

A strong absorption band assigned to the Amide I region appears at 1630 cm^−1^ for gelatin, 1637 cm^−1^ for whey, 1639 cm^−1^ for WGeGly, 1637 cm^−1^ for WGRCE and 1639 for WGRCEt [28]. This band is predominantly attributed to C=O stretching vibrations of the peptide backbone [45] and is highly sensitive to changes in protein secondary structure and intermolecular interactions. The shift in amide I toward higher wavenumber after thermal treatment indicated a consolidation of triple helix structures due to the renaturation of protein structure of proteins, well correlated with the XRD measurements that indicated the increase in crystallinity. Minor shifts in the Amide I band position indicate modifications in the local chemical environment of the protein chains due to interactions with glycerol and red cabbage extract.

The Amide II band, originating mainly from N–H bending and C–N stretching vibrations, is observed at 1516 cm^−1^ for gelatin, 1524 cm^−1^ for whey, and shifts toward higher wavenumbers (1539 cm^−1^) in the glycerol-containing samples (WGeGly), 1554 in WGRCE, and 1540 in WGRECt. This shift suggests specific interactions between glycerol molecules and RCE and the protein matrix, affecting the vibrational behavior of peptide bonds.

The introduction of glycerol and RCE determined a shift in the amide III band toward higher values of wavenumbers, while thermal treatment had a minor influence on the position of the amide III band.

In the lower wavenumber region, bands around 1070–1080 cm^−1^ are detected in all samples and can be attributed to C–O and C–N stretching vibrations from the protein backbone, with contributions from glycerol. Additionally, a distinct and intense band at approximately 1030 cm^−1^ is associated with C–O stretching vibrations of primary hydroxyl groups of glycerol, confirming its effective incorporation as a plasticizer within the hydrogel network.

Although no new absorption bands are observed in the samples containing red cabbage extract, noticeable changes in band intensity, position, and symmetry—particularly in the Amide I and Amide II regions—indicate strong non-covalent interactions, mainly hydrogen bonding, between the protein matrix, glycerol, and bioactive compounds from the extract. These interactions are expected to influence protein chain organization and secondary structure, which may subsequently affect the physicochemical properties and pH-responsive behavior of the hydrogels.

The FT-IR results are in good agreement with the structural parameters derived from XRD analysis. The increased intensity of the Amide A band in the WGRCE sample, associated with enhanced hydrogen bonding, correlates with the lowest degree of crystallinity (5.8%) and the broad amorphous diffraction peak observed in XRD, indicating a highly disordered hydrogel network.

Minor shifts and changes in the symmetry of the Amide I and Amide II bands reflect conformational rearrangements of the protein chains induced by glycerol and red cabbage extract. These spectral modifications are consistent with the reduction or disappearance of helicoidal ordering detected by XRD, particularly in the WGeGly sample, where no helicoidal diffraction peak and zero crystallinity were observed.

Furthermore, the reappearance of helicoidal diffraction and the increased crystallinity degree in the thermally treated WGRCEt sample (25.3%) suggest partial structural reorganization, which is also reflected in the stabilization of amide band positions in the FT-IR spectra. Overall, the combined FT-IR and XRD analyses confirm that the interactions between proteins, glycerol, and bioactive compounds are predominantly non-covalent and lead to significant modifications of protein secondary structure and long-range ordering, directly influencing the pH-responsive behavior of the hydrogels.

In the FTIR spectrum of the Red Cabbage Extract (RCE) gel, obtained following the drying of RCE, the broad absorption band centered at 3261 cm^−1^ is assigned to the stretching vibrations of hydroxyl groups (–OH) [34], intensified by the hydrophilic nature of the polysaccharide matrix. The peaks at 2928 and 2874 cm^−1^, along with the shoulder at 2781 cm^−1^, are attributed to both aliphatic and aromatic C-H stretching vibrations [18,23].

The prominent, wide absorption band between 1725 and 1500 cm^−1^ arises from the overlap of the C=C stretching of the aromatic rings [18,23], –OH bending (water/phenols), and C=O vibrations. Notably, the absence of a distinct peak at 1710 cm^−1^ suggests that the carboxylic groups are not in a free state; instead, they are likely involved in strong intermolecular hydrogen bonding or electrostatic interactions within the pectin-rich gel matrix.

The complex fingerprint region between 1494 and 1192 cm^−1^ reflects the presence of carbohydrates, primarily pectin. Within this range, the peak at 1396 cm^−1^ is attributed to the in-plane bending of O–H [5] and the symmetrical stretching of the carboxylate ion (—COOH^−^), confirming the ionized state of organic acids. The shoulder at 1450 cm^−1^, due to CH_2_ and CH_3_ and scissoring, further corroborates the presence of the aromatic rings and the aliphatic chains of the saccharide units. The peak at 1329 cm^−1^ corresponds to the skeletal stretching of C–O bonds from the anthocyanin aromatic rings, combined with C–H and O–H vibrations from the pyran structure of the anthocyanins’ central ring [5]. The band at 1261 cm^−1^ (often merging in this region) relates to the C–O–C stretching of the glycosidic linkages. Finally, the intense peak at 1027–1021 cm^−1^ is characteristic of C–O stretching vibrations in alcohols (secondary) and ethers, typical for the flavonoid molecules and the sugar moieties (pyranose rings) present in the extract [46].

The combined FT-IR and XRD analyses provide complementary evidence of the structural reorganization induced by glycerol and red cabbage extract (RCE) within the gelatin–whey hydrogel matrix. The increased intensity of the Amide A band in the WGRCE sample, associated with enhanced hydrogen bonding interactions between anthocyanin polyphenols and protein chains, correlates well with the broad amorphous diffraction halo and the lowest crystallinity degree (5.8%) observed by XRD, indicating a more disordered yet interaction-rich network. The absence of new FT-IR bands and the presence of shifts in the Amide I and II regions confirm that these interactions are predominantly non-covalent, mainly hydrogen bonding, which modifies the local secondary structure without disrupting the integrity of the protein backbone. This structural modulation is further supported by the disappearance of helicoidal ordering in the WGeGly sample and its partial recovery in the thermally treated WGRCEt hydrogel, where XRD revealed increased crystallinity (25.3%) accompanied by stabilization of amide band positions in FT-IR spectra.

Such interplay between short-range molecular interactions (FT-IR) and long-range structural organization (XRD) demonstrates that RCE acts not merely as a colorant but as an active component capable of participating in the protein network through hydrogen bonding. The resulting semi-amorphous yet cohesive architecture favors controlled vapor diffusion and reversible proton exchange, essential features for reliable pH-responsive sensing. Therefore, the incorporation of RCE contributes both chromatic functionality and beneficial structural modulation of the hydrogel matrix, reinforcing its suitability as a bioactive pigment for intelligent packaging applications.

### 2.6. TVB-N Evolution During Chicken Meat Storage

**Figure 7:**
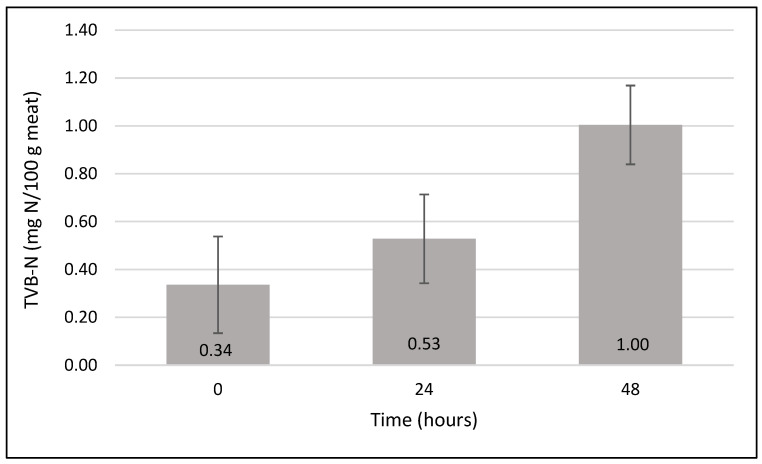
Total volatile basic nitrogen evolution versus time during poultry meat storage at room temperature (23 °C).

The variation in total volatile basic nitrogen (TVB-N) was monitored in chicken meat samples stored at room temperature (23 °C) and in the same packaging conditions as those used for the colorimetric evaluation of the hydrogel indicators. As shown in Figure 7, TVB-N values increased progressively during storage, reflecting the accumulation of volatile basic compounds generated during protein degradation. At the initial stage (0 h), the TVB-N content remained low, corresponding to the freshness state of meat. After 24 h of storage, a noticeable increase in TVB-N was observed (0.53 mg N/100 g meat ± SD), indicating the onset of spoilage processes. A further increase up to 1 mg N/100 g meat ± SD was recorded at 48 h, confirming the progressive deterioration of the meat matrix. This trend was consistent with the colorimetric changes observed for the RCE-loaded hydrogel indicators, which exhibited increasing ΔE_00_ values over the same storage period. The correlation between the increase in TVB-N and the hydrogel color variation supports the capability of the proposed indicator to respond to volatile amines generated during poultry spoilage under real packaging conditions.

## 3. Conclusions

Gelatin–whey hydrogels incorporating red cabbage extract were successfully fabricated and characterized. XRD analyses confirmed predominantly amorphous protein lattice with short-range β-sheet ordering, while RCE incorporation and mild thermal treatment promoted further homogeneity and a higher crystallinity level. SEM revealed a dual structure of compact surface domains and porous inner layers, regulating gas diffusion and enabling controlled color response.

This dual-layer structure—with a dense surface and porous interior—could serve as a tunable platform for designing time–temperature or spoilage indicators featuring delayed activation or staged response behavior, tailored to the requirements of cold-chain food logistics. The dual microstructure revealed by SEM, consisting of a compact surface layer and a porous internal network, appears to play an important role in regulating volatile amine diffusion, enabling a gradual and stable colorimetric response of the hydrogel indicator.

The FT-IR results are consistent with the structural characteristics revealed by XRD analysis.

A clear correlation was observed between the measured color difference (ΔE_00_) and estimated pH, with significant chromatic transitions (ΔE_00_ > 5) occurring for pH variations as small as 0.5–1 units. This confirms the hydrogel’s high responsiveness to chemical changes in the surrounding environment and supports its use for real-time, non-invasive freshness monitoring. The gelatin–whey hydrogel incorporating red cabbage extract exhibited a stable colorimetric response within the pH range 4.5–7.5, matching the spoilage-related pH window of fresh poultry and red meat. This range confirms their suitability as pH-sensitive freshness indicators for intelligent packaging applications.

Overall, these findings highlight the strong relationship between the structural organization of the hydrogel matrix and its pH-responsive colorimetric performance, supporting its potential for intelligent food packaging applications.

Future work will focus on integrating the hydrogel indicators into real-time monitoring systems. Planned studies include UV–VIS measurements performed directly on the hydrogel patches inside the meat packages during storage, to establish quantitative relationships between optical and spoilage markers. In addition, a mobile phone application will be developed to automatically detect color shifts and estimate the degree of freshness, enabling practical and consumer-accessible use of these intelligent hydrogel sensors in food packaging.

It should be noted that the colorimetric response of anthocyanin-based indicators is primarily associated with the accumulation of volatile basic compounds, such as ammonia and biogenic amines, generated during protein degradation. Consequently, although microbial activity may begin earlier during meat storage, significant chromatic transitions generally occur once these volatile compounds reach detectable levels. Therefore, the developed hydrogel system is particularly sensitive to intermediate and advanced stages of spoilage, where the accumulation of alkaline volatiles induces measurable pH shifts and visible color changes.

## 4. Materials and Methods

### 4.1. Materials

Whey protein isolate (W), produced by Carbery Group Carbery (Cork, Ireland) and supplied by S.C. Way Better Nutrition from Cluj-Napoca, Gelatin Reagent (AMRESCO, LLC, Fountain Parkway Solon, Cleveland, OH, USA), and glycerol (Sigma-Aldrich, Taufkirchen, Germany) were used as precursors for the hydrogel preparation. The red cabbage (*Brassica oleracea* var. *capitata f. rubra*) was purchased from a local agricultural market (Cluj-Napoca, Romania) and used as the source of anthocyanin-rich extract (RCE).

Buffer solutions with pH values ranging from 4.5 to 7.5 were prepared using analytical grade reagents (Sigma-Aldrich, Taufkirchen, Germany): acetic acid/sodium acetate (pH 4.5–5.5) and sodium dihydrogen phosphate/disodium hydrogen phosphate systems (pH 6.0–7.5) according to standard buffer preparation protocols.

The chemicals employed for the extraction of volatile nitrogen compounds from the chicken meat samples included acetic acid (10% *v*/*v*), respectively, boric acid (2% *w*/*v*) (Chempur, Piekary Slaskie, Poland) as the absorbing solution in the central compartment of the microdiffusion system. Potassium carbonate (K_2_CO_3_) (Chimopar, București, Romania) was applied as an alkaline agent to release volatile bases from the extract, while hydrochloric acid (HCl 0.01 *N*) (Chempur, Piekary Slaskie, Poland) served as the titrant for the quantitative determination of total volatile basic nitrogen (TVB-N). A mixed indicator composed of methyl red (Chinoin, Budapest, Hungary) and bromcresol green (Nordic Chemicals, Cluj-Napoca, Romania) was used to visually detect the endpoint of the titration. The indicator solutions were prepared as 0.1% (*w*/*v*) dissolved in concentrated ethanol, and subsequently mixed in equal proportions to obtain the mixed indicator solution.

### 4.2. Hydrogel and Red Cabbage Extract Preparation

A hydrogel formulation was developed using whey powder in a 5% (*w*/*w*) aqueous solution [27,28]. Whey powder was dissolved in distilled water (22.8 ± 0.3 °C) and homogenized using a magnetic stirrer for 2 h (≈23 °C) to ensure complete solubilization. Following the 2 h magnetic stirring process, the remaining components—gelatin, glycerol, and red cabbage extract (RCE)—were incorporated into the whey solution under the same temperature conditions, and the mixture was continuously stirred until homogeneous. Due to the low liquid volume, the final mixture was gently heated in a water bath at 50 °C to facilitate complete gelatin dissolution. The pH of the hydrogel solution was 5.0 before drying. The homogeneous solution was then poured into silicone molds and subjected to drying in a ventilated oven at 25 °C until drying. The specific concentrations of each component used in the hydrogel formulation are detailed in Table 2.

The red cabbage extract (RCE), serving as a natural source of anthocyanins, was obtained through a multi-step process [11]. Fresh red cabbage leaves with intense purple pigmentation were selected, avoiding the central rib areas. The leaves were finely grated using a small-hole kitchen grater to increase the exposed surface area and facilitate the subsequent extraction. The grated material was subjected to microwave heating at medium power (≈600 W) for about 20 min using a domestic microwave oven, Vortex (distributed by Complet Electro Serv S.A., Cluj-Napoca, Romania), operated on the second heating level to soften the tissue and partially rupture cell walls. The softened plant material was then infused in distilled water at room temperature (≈23 °C) for 60 min, in the absence of daylight, to promote the release of anthocyanins and other phenolic compounds. The mixture was filtered through fine gauze to remove solid residues, and the resulting anthocyanin-rich extract was stored in an amber glass bottle at 4 °C, protected from light, until further use in hydrogel preparation.

The total anthocyanin content was determined using the pH differential method described by [47] which is based on the structural transformation of anthocyanin pigments as a function of pH, resulting in a measurable change in absorbance. The pH-differential method was selected due to its high specificity for flavylium/hemiketal interconversion and its widespread use as a reference technique for quantifying total monomeric anthocyanins in food-derived extracts.

The sample was diluted 20-fold with each buffer, and the absorbance was measured at 520 nm and 700 nm using a Lambda 35 UV-Vis spectrophotometer (Perkin Elmer, Waltham, MA, USA) (1 cm path length). The corrected absorbance (*A*) was calculated according to the equation Giusti and Wrolstad [47]:

(1) $$A=\left(A520-A700\right)pH1.0-\left(A520-A700\right)pH4.5$$

The total anthocyanin concentration, expressed as mg cyanidin-3-glucoside equivalents per liter (mg C3G/L), was calculated using the following formula:

(2) $$C=A\times MW\times DF\times \frac{1000}{\varepsilon \times l}$$

where *MW* = 449.2 g · mol^−1^ molecular weight of cyanidin-3-glucoside, *DF* = 20 dilution factor, ε = 26.900 L · mol^−1^ · cm^−1^ molar extinction coefficient, *l* = 1 cm (path length).

The concentration of total anthocyanin content (as Cyanidin-3-glucoside equivalents) in red cabbage extract was 18.3687 mg/L.

### 4.3. Colorimetric pH Sensitivity Assessment

The most commonly adopted method, also employed by other authors [48], to evaluate the pH responsiveness of hydrogel films, is to prepare a series of buffer solutions with pH values of 4.5, 5.0, 5.5, 6.0, 6.5, 7.0, and 7.5, in order to be within the pH range of fresh meat, which varies from 5.4 to 5.8 for beef, from 5.5 to 5.8 for pork, and from 5.8 to 6.0 for poultry. Strips of dried hydrogel films were immersed in each buffer solution and allowed to equilibrate. Color changes were visually evident and documented for each pH condition, using a smartphone camera under consistent lighting conditions. The images were subsequently uploaded to the web-based platform https://imagecolorpicker.com/ (accessed on 12 December 2025) to extract color values; therefore, for each pH level, the corresponding HEX and RGBA color codes were recorded.

**Figure 8:**
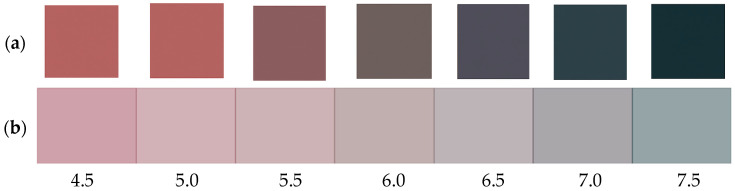
(**a**) Colorimetric scale showing pH-dependent color transitions of whey–gelatin hydrogel enriched with anthocyanins from red cabbage extract. (**b**) Simulated colorimetric scale with 50% transparency.

To illustrate the colorimetric sensitivity of the hydrogel to pH variations, narrow strips of the hydrogel containing RCE were immersed in a series of buffer solutions with pH from 4.5 to 7.5, at 0.5 unit intervals [34]. After equilibration, the color of each strip was recorded and analyzed. The hydrogel exhibited a gradual color transition from deep red under acidic conditions to bluish-green in mildly basic environments, indicating the characteristic chromatic shift in anthocyanin pigments. Based on captured colors, a visual color scale was constructed by extracting the corresponding color codes (HEX and RGBA) for each pH value, as shown in Figure 8a. To simulate the visual appearance of the semi-transparent hydrogel films, the extracted color codes (HEX) were converted to RGBA format, and a 50% transparency level (alpha = 0.5) was digitally adjusted, as can be seen in Figure 8b. This visualization was generated using Python 3.10 and the Matplotlib library 3.7 enabling a closer approximation of the film’s actual appearance under standardized lighting and a white matte background.

### 4.4. Application of Hydrogel Films to Monitor Chicken Meat Spoilage

**Figure 9:**
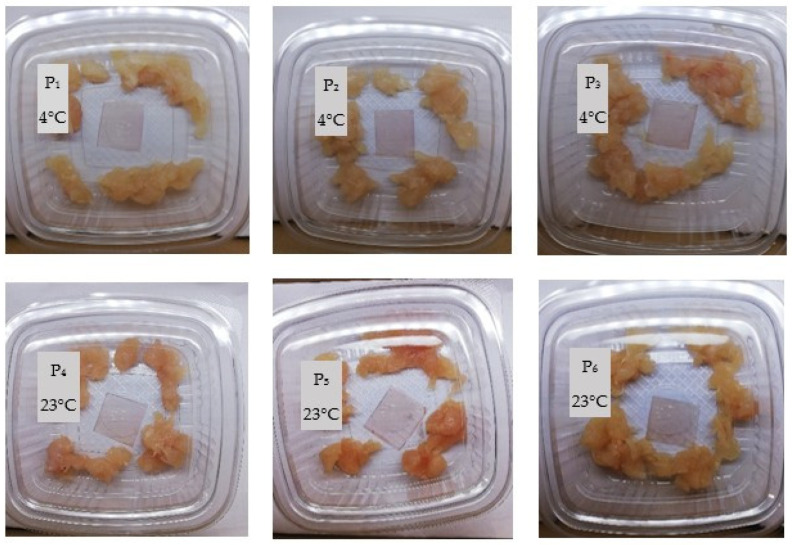
Packaging system comprising polypropylene containers with minced poultry meat and protein-based hydrogels (gelatin-whey) functionalized with RCE.

Approximately 50 g of fresh chicken breast meat was finely chopped using a kitchen knife and placed into transparent, colorless polypropylene (PP) containers, as illustrated in Figure 9. A small square piece of dried hydrogel film was attached to the inside of each container lid using a drop of gelatin solution. The containers were then sealed.

Two storage conditions were tested:

- Three samples were kept at room temperature (≈23 °C)
- Three samples were stored under refrigeration (4 ± 1 °C)

The colorimetric response of the hydrogel indicators was monitored over time. Photographs were taken every 12 h to capture the color changes.

To ensure consistent and accurate photographic documentation, a custom light-controlled setup was used, and samples were placed inside a cardboard box. A uniform LED strip was mounted across the top interior of the box to provide even illumination, minimizing the influence of external light sources. A white sheet of paper was placed at the base of the box to serve as a neutral background, and each meat sample was positioned on this surface during image acquisition. All photographs were taken using the same smartphone camera throughout the entire experiment. The closed imaging box ensured constant illumination and minimized external light variability, improving the reproducibility of the RGB color extraction.

### 4.5. Color Changes Determination

The samples used for meat monitoring (P_1_–P_6_) were prepared according to the WGRCE formulation described in Table 2. Six hydrogel samples were analyzed based on photographs acquired according to the procedure described in Section 4.3. Samples P_1_–P_3_ were stored under refrigerated conditions (4 ± 1 °C), while samples P_4_–P_6_ were kept at room temperature (≈23 °C). The reference point for color comparison was defined at 12 h, as initial changes are primarily attributed to moisture absorption rather than volatile amine reactions. All colorimetric measurements were performed in triplicate (*n* = 3) to ensure statistical relevance. Colorimetric analysis was performed by image-based evaluation of the average RGB values converted into the CIELAB color space [49]. Color differences were quantified using the ΔE_00_ parameter, calculated according to the CIEDE2000 formula, relative to the reference sample at 12 h. ΔE_00_ values greater than 5 were considered visually perceptible.

The RGB values extracted from the photographic dataset were converted into the CIELAB color space using the standard sRGB–XYZ–CIELAB transformation pipeline under D65 illumination conditions. This image-based colorimetric approach is widely applied for the quantitative evaluation of chromatic changes in intelligent food packaging materials and pH-sensitive systems. Color differences were calculated using the CIEDE2000 (ΔE_00_) formula, which provides improved perceptual uniformity compared to earlier color difference metrics, particularly for low-chroma and purple-to-blue color transitions characteristic of anthocyanin-based materials [11].

### 4.6. Morphology of Whey and Gelatin Hydrogel with Red Cabbage Extract by SEM

The microstructure and morphology of the hydrogel based on whey and gelatin, and improved with red cabbage extract, was studied with the help of an Inspect-S Scanning Electron Microscope (FEI Company, Hillsboro, OR, USA). Samples were sputter-coated with 10 nm AU (Agar Automated Sputter Coater, Essex, UK) prior to observation. The coating was performed at a sputtering current of 20 mA for 90 s under a working pressure of approximately 0.05 mbar. SEM imaging was carried out in low-vacuum mode (LBT) at an accelerating voltage of 30 kV and working distance of 18.1 mm, with an emission current of about 100 µA and an observation chamber pressure of 10^−2^ mbar. Surface and cross-sectional images were captured using an HR-STEM Hitachi SU8230 microscope (Hitachi High-Technologies Corporation, Tokyo, Japan) equipped with a secondary electron detector (SE mode) to highlight topographic contrast.

### 4.7. Structural Analysis of Precursors and Hydrogel Samples by X-Ray Diffraction

The X-ray diffraction of the precursors and whey-and-gelatin-based hydrogels, improved and functionalized with RCE, was performed with the XRD-6000 SHIMADZU (Kyoto, Japan) diffractometer with CyKα radiation (λ = 1.54056 Å) and 2*θ* angle range between 3° and 80°.

The interplanar distance *d* (Equation (3)) corresponding to the diffraction peaks was calculated using Bragg’s law:

(3) $$d_{h,k,l}=\frac{n\lambda }{2sin\theta }(Å)$$

where: *n* represents the diffraction order, the value of which is 1 for most of the cases; *θ* is the Bragg angle, *λ* is the wavelength of the radiation used for the diffraction, and *h*, *k*, *l* represent the Miller indices associated with the specific crystallographic planes.

The degree of crystallinity was estimated using the integration method, determined by the following equation:

(4) $$CI\left(\%\right)=\frac{A_{helical}}{A_{total}}\times 100$$

where: *A_helical_* is the area of the peak corresponding to the triple-helix structure (2*θ* < 13°) and *A_total_* represents the total area under the diffraction curve in the range of 4–40°.

The Full Width at Half Maximum (FWHM) of the diffraction peaks was determined to evaluate the peak broadening and to compare the degree of structural order among the hydrogel samples.

### 4.8. FT-IR Spectral Analysis

Fourier transform infrared spectroscopy (FT-IR) was conducted to analyze the chemical nature of the nanoparticle surfaces using a Shimadzu IR-Prestige FTIR Spectrometer equipped with a diamond PIKEMIRacle single reflection plate unit. The spectra were taken in the 600–4000 cm^−1^ range with a resolution of 4 cm^−1^.

Generative artificial intelligence (GenAI), specifically a large language model (ChatGPT, CPT-4, developed by OpenAI), was employed during the preparation of this manuscript to assist authors in several stages of scientific communication. The tool was used to support the interpretation and structuring of experimental results, including data derived from colorimetric analysis (ΔE_00_), and pH response of the hydrogel system. The study design, experimental procedures, data collection, and initial analysis were conducted entirely by the authors. The AI tool was not used for data generation or statistical computation. Its use was limited to enhancing the quality of scientific writing and facilitating the communication of complex findings in a concise and consistent manner.

### 4.9. Determination of Total Volatile Basic Nitrogen Analysis (TVB-N) by Modified Conway Microdiffusion Method

Total volatile basic nitrogen (TVB-N) was determined using a modified Conway microdiffusion method [50,51]. Chicken breast samples stored at room temperature (23 °C) in the same packaging conditions as those used in the colorimetric assay were analyzed at 0.24 and 48 h, with three independent replicates for each time point. Briefly, 10 g of ground chicken breast was homogenized with 90 mL of 10% acetic acid and filtered. In the microdiffusion system, 2 mL of 2% boric acid containing a mixed indicator (methyl red and bromcresol green) was placed in the central compartment, while 2 mL of filtrate and 1 mL of saturated K_2_CO_3_ were added to the outer compartment. The sealed system was incubated at 37 °C for 90 min. After diffusion, the boric acid solution was quantitatively transferred into an Erlenmeyer flask, the compartment was rinsed with 5 mL of distilled water, and the combined solution was titrated with 0.01 N HCl until the endpoint color changed from green to pink. A blank was run under identical conditions. TVB-N values were expressed as mg N/100 g meat according to the following Equation (5).

(5) $$TVB-N(mg\;N/100\;g\;meat)=\frac{\left(V_{s}-V_{b}\right)\times N\times 14.007}{m}\times 100$$

where *V_s_* is the volume (mL) of HCl used for titration of the sample, *V_b_* is the volume (mL) of HCl used for the blank determination, *N* is the normality of the HCl solution (0.01 N), and *m* represents the mass (g) of the meat sample. The factor 14.007 corresponds to the atomic mass of nitrogen.

## Figures and Tables

**Table 1:** Structural parameters derived from XRD analysis for the investigated precursors and hydrogel samples.

Sample	2θ Amorphous (Degree)	d Amorphous (Å)	FWHM_1_ (Degree)	2θ Helix (Degree)	d Helix (Å)	FWHM_2_ (Degree)	Degree of Crystallinity (%)
Whey	18.95	4.68	6.90	8.36	10.56	2.92	40.5
Gelatin	19.11	4.64	11.44	7.89	11.19	3.56	10.9
WGRCE *	20.22	4.39	6.99	7.50	11.78	3.86	5.8
WGRCEt **	19.92	4.45	6.90	7.20	12.27	3.20	25.3
WgeGly ***	19.35	4.58	6.83	-	-	-	-

* WGRCE: hydrogel with RCE, dried at room temperature (23 °C); ** WGRCEt: hydrogel containing RCE, further thermally treated at 50 °C for 3 days after drying at 25 °C; *** WGeGly: control hydrogel (gelatin–whey–glycerol, no RCE).

**Table 2:** Composition and relative proportions of components used in hydrogel formulation.

Whey (%)	Glycerin (%)	Gelatin (%)	Red Cabbage Extract (%)	Distilled Water (%)	Total (%)
3.03	3.03	6.06	30.30	57.58	100

Concentrations are expressed as weight ratios relative to total formulation mass (*w*/*w*).

## Data Availability

The original contributions presented in this study are included in the article/supplementary material. Further inquiries can be directed to the corresponding author.

## Supplementary Materials

The following supporting information can be downloaded at: https://www.mdpi.com/article/10.3390/gels12040292/s1, Figure S1. Comparison of the colorimetric response of red cabbage extract–loaded hydrogel samples stored under refrigerated conditions and at room temperature. Marked differences were observed between the two storage conditions, with negligible color changes under refrigeration and pronounced chromatic variations at room temperature.

## Abbreviations

The following abbreviations are used in this manuscript:

C3G	Cyanidin-3-glucoside
CIEDE2000	International Commission on Illumination Delta E 2000
FWHM	Full Width at Half Maximum
Ge	Gelatin
Gly	Glycerol
RCE	Red Cabbage Extract
SD	Standard deviation
TVB-N	Total Volatile Basic Nitrogen
W	Whey protein isolate
WGeGly	Whey and gelatin-based hydrogel with glycerol as plastifier
WGRCE	Whey and gelatin-based hydrogel functionalized with red cabbage extract (RCE)
WGRCEt	Whey and gelatin-based hydrogel functionalized with red cabbage extract (RCE) subjected to post-synthesis drying at 50 °C for 3 days
XRD	X-ray Diffraction
