# Supplementary material for: Gelatin Hydrogel pH Sensors Incorporating Anthocyanins for Intelligent Food Packaging: Towards Enhanced Food Spoilage Detection
Source: Gels. 2026 Mar 31;12(4):292. doi: 10.3390/gels12040292 (PMC13116531; doi:10.3390/gels12040292)
Supplement: Supplementary file 1 [file gels-12-00292-s001.zip › gels-4092034-supplementary.pdf]

Supplementary Materials

Figure S1

Comparison of the colorimetric response of red cabbage extract–loaded hydrogel samples stored under refrigerated conditions and at room temperature. Marked differences were observed between the two storage conditions, with negligible color changes under refrigeration and pronounced chromatic variations at room temperature.

Time [hours]	P_1	P_2	P_3	P_4	P_5	P_6
0	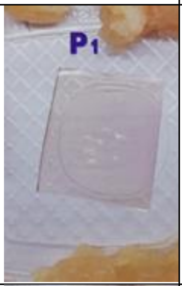	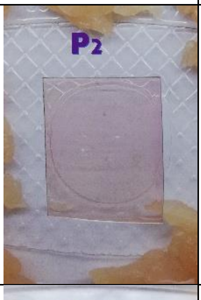	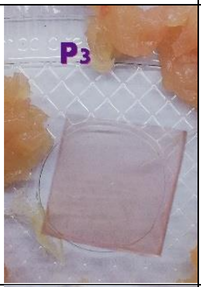	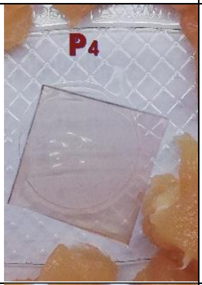	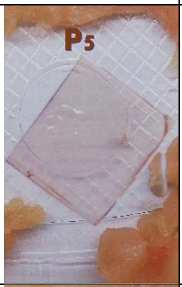	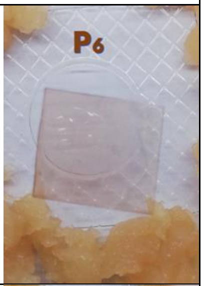
12 h	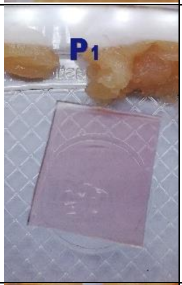	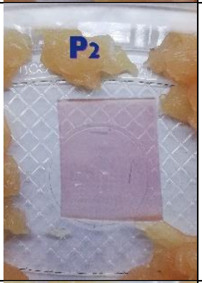	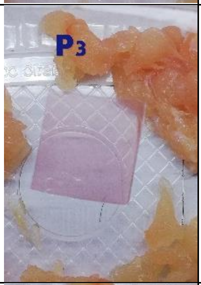	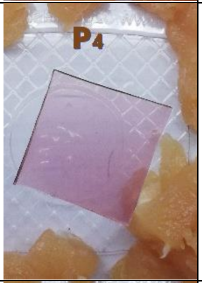	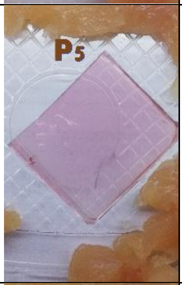	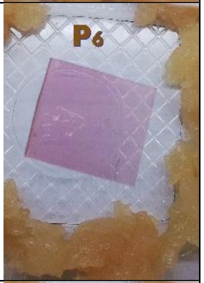
24 h	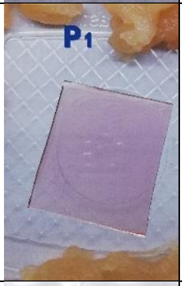	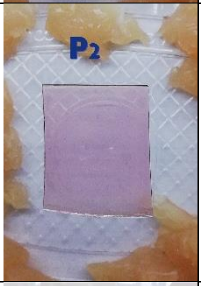	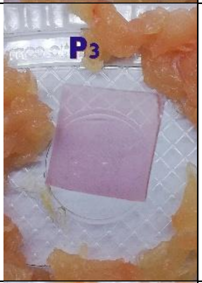	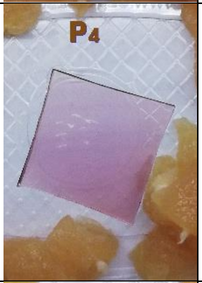	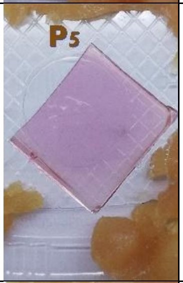	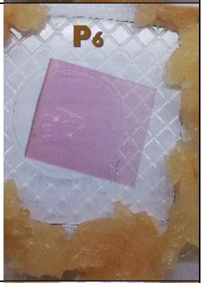
36 h	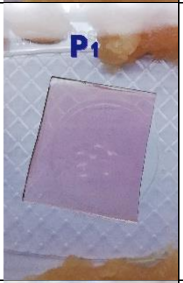	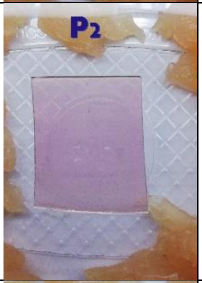	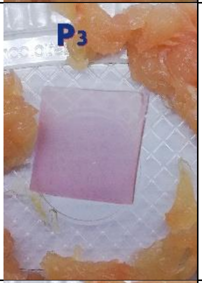	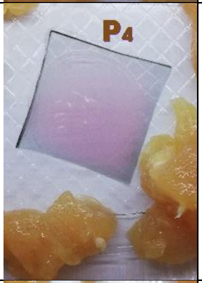	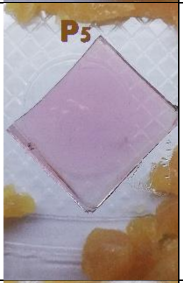	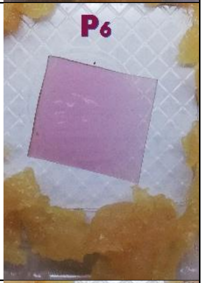
48 h	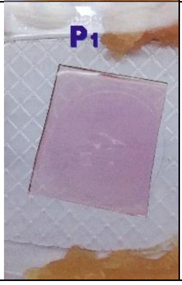	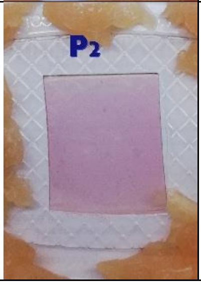	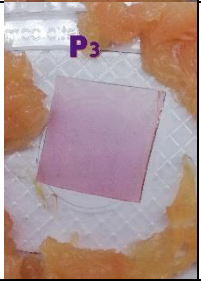	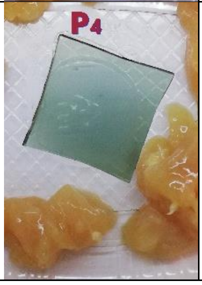	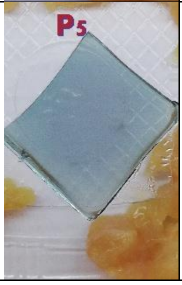	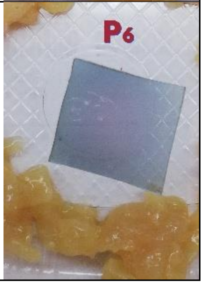

60 h	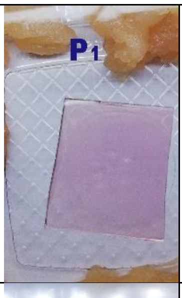	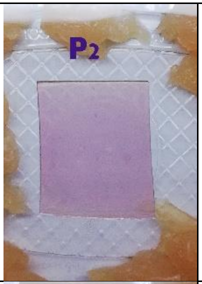	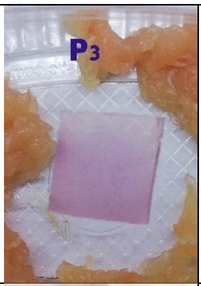	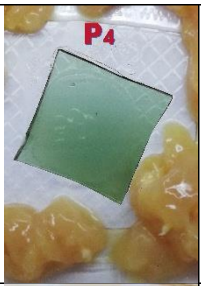	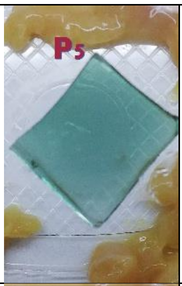	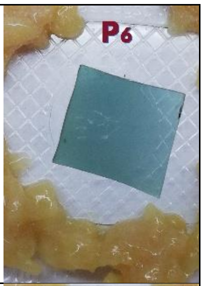
72 h	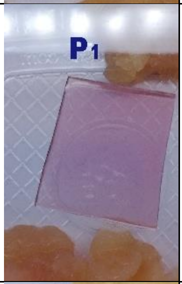	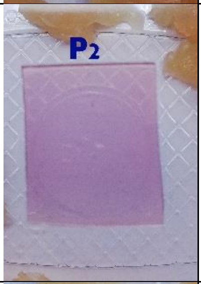	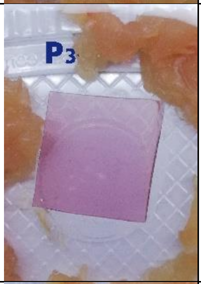	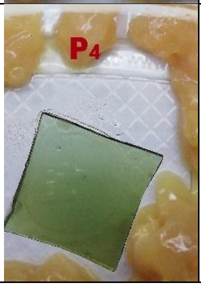	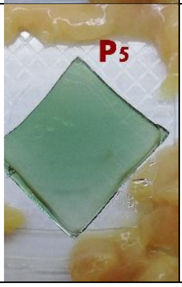	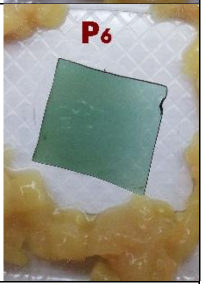
84 h	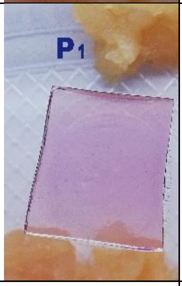	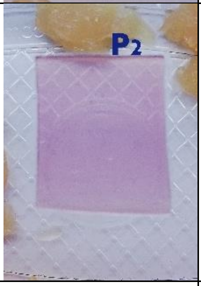	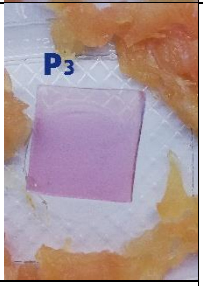	We took no more pictures because the meat degraded and began to smell very bad.		
96 h	No color change	No color change	No color change			
108 h	No color change	No color change	No color change			
120 h	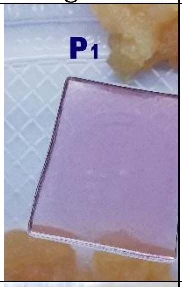	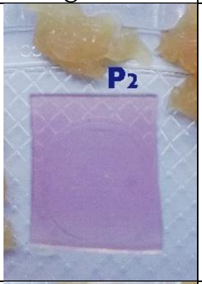	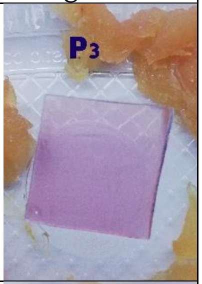			
132 h	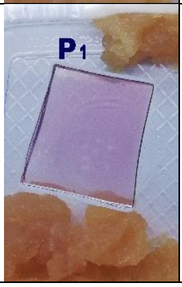	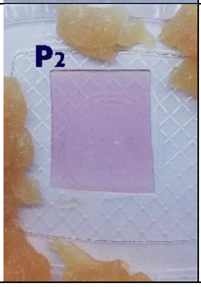	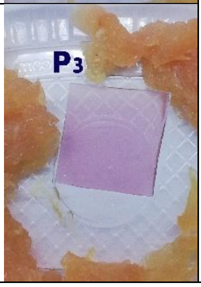			
